# Sternocleidomastoid Muscle Flap in an Older Patient With Congenital Muscular Torticollis

**DOI:** 10.7759/cureus.58517

**Published:** 2024-04-18

**Authors:** Naoki Matsuura, Reiko Asato, Shohei Ishihara, Rikako Matsuura, Yusuke Shimizu

**Affiliations:** 1 Plastic and Reconstructive Surgery, University of the Ryukyus Hospital, Nishihara, JPN

**Keywords:** flap reconstruction, sternocleidomastoid muscle, muscle flap, older patients, congenital muscular torticollis

## Abstract

Congenital muscular torticollis (CMT) is caused by muscle damage during childbirth, tumors, or masses within the muscles and generally resolves with physical therapy during infancy. In this study, we performed reconstruction after resection of a parotid gland tumor using a sternocleidomastoid muscle (SCMM) flap in an older patient with neglected CMT. The patient was a 64-year-old woman who had had a left-sided oblique neck since infancy but had never received any treatment, including physical therapy. She underwent parotid tumor resection and SCMM flap transfer. The SCMM flap can be safely elevated using indocyanine green fluorescence angiography, with the middle pedicle serving as the feeding vessel to fill the parotid defect. Three months after surgery, the torticollis had improved and the cheek depression was not noticeable, indicating the effectiveness of surgical treatment for CMT in older patients and the possibility of using SCMM as a muscle flap.

## Introduction

Congenital muscular torticollis (CMT) is the third most common congenital musculoskeletal anomaly, following hip dislocation and clubfoot [1]. CMT is caused by muscle damage during childbirth, tumors, or masses within muscles [2], with the sternocleidomastoid muscle (SCMM) being the most commonly affected [3]. It is established that more than 90% of cases experience relief or improvement through physical therapy during infancy, underscoring the significance of early medical intervention postdiagnosis [4,5]. However, there have been reports on the effectiveness of surgical treatment in cases where improvement was not observed with physical therapy or in cases that did not receive early medical intervention, such as physical therapy [6-8].

The SCMM flap is effective in preventing Frey’s syndrome during parotid tumor resection (shallow lobectomy, total resection, or extended total resection). It improves cosmetic outcomes by preventing facial depression through the filling of thick tissue [9] and serves as preparation for a nerve graft bed when a nerve graft is performed [10].

To our knowledge, no prior studies have utilized SCMM muscle flaps, responsible for CMT, to achieve both parotidectomy defect reconstruction and torticollis correction in a single procedure and/or in the elderly.

Here, we present a case in which SCMM flap elevation after parotid tumor resection in an older patient with untreated CMT improved postoperative cheek depression, prevented Frey’s syndrome, and improved the torticollis all through a single procedure.

## Case presentation

Here, we present the case of a 64-year-old woman who had been experiencing CMT since infancy but had never received any treatment, including physical therapy. The patient initially presented to the otorhinolaryngology department with swelling near the lower part of the left ear and left facial nerve palsy.

**Figure 1:**
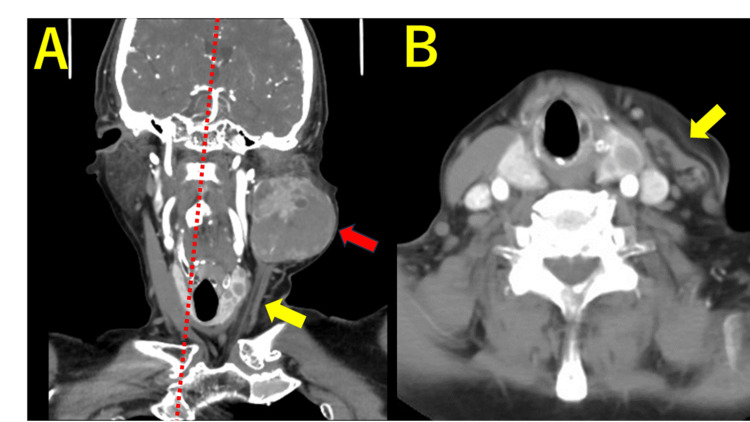
Preoperative CT images (A) Coronal section. A left parotid tumor is observed (red arrow). Atrophy of the left SCMM (yellow arrow) and oblique neck (red dotted line) are noted. (B) Axial section. Atrophy of the left SCMM (yellow arrow) is noted. SCMM, sternocleidomastoid muscle

Contrast-enhanced CT revealed a parotid tumor (Figure 1). Based on the preoperative CT images and facial nerve paralysis, malignancy was clinically suspected; however, the histopathological examination of biopsy tissue showed no evidence of malignancy. Moreover, rapid intraoperative pathology revealed a benign tumor; therefore, the head and neck surgeon completed a superficial lobectomy of the left parotid gland through an S-shaped incision. Following the parotid tumor lobectomy, the patient was referred to the plastic and reconstructive surgery department for reconstruction of the defect. Preoperative contrast-enhanced CT showed a partially scarred and thinning left SCMM that had been compressed by the parotid tumor, with some remaining muscle tissue. Consequently, we opted for parotid defect reconstruction using an SCMM flap.

**Figure 2:**
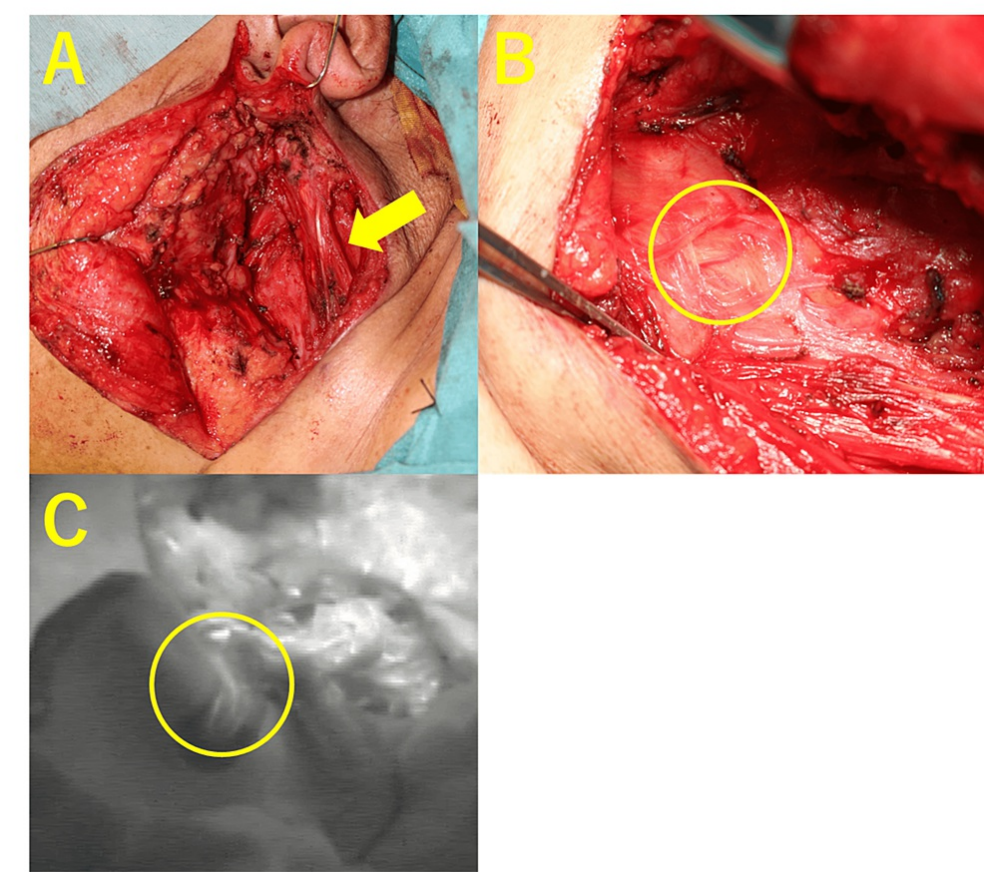
Intraoperative image (A) Findings of a resected parotid tumor. Atrophy of the left SCMM is shown (yellow arrow). (B) Middle pedicle flow from the back surface of the SCMM is observed (yellow circle). (C) The ICG fluorescence contrast scan also confirms that the muscle is stained from the middle pedicle (yellow circle). ICG, indocyanine green; SCMM, sternocleidomastoid muscle

After tumor resection, a banded SCMM was observed, which was dissected in a bipolar manner, both distal and proximal to the muscle body. A blood vessel was observed flowing into the remaining central part of the SCMM, and indocyanine green (ICG) fluorescence contrast showed staining of the muscle from the vessel (Figure 2).

**Figure 3:**
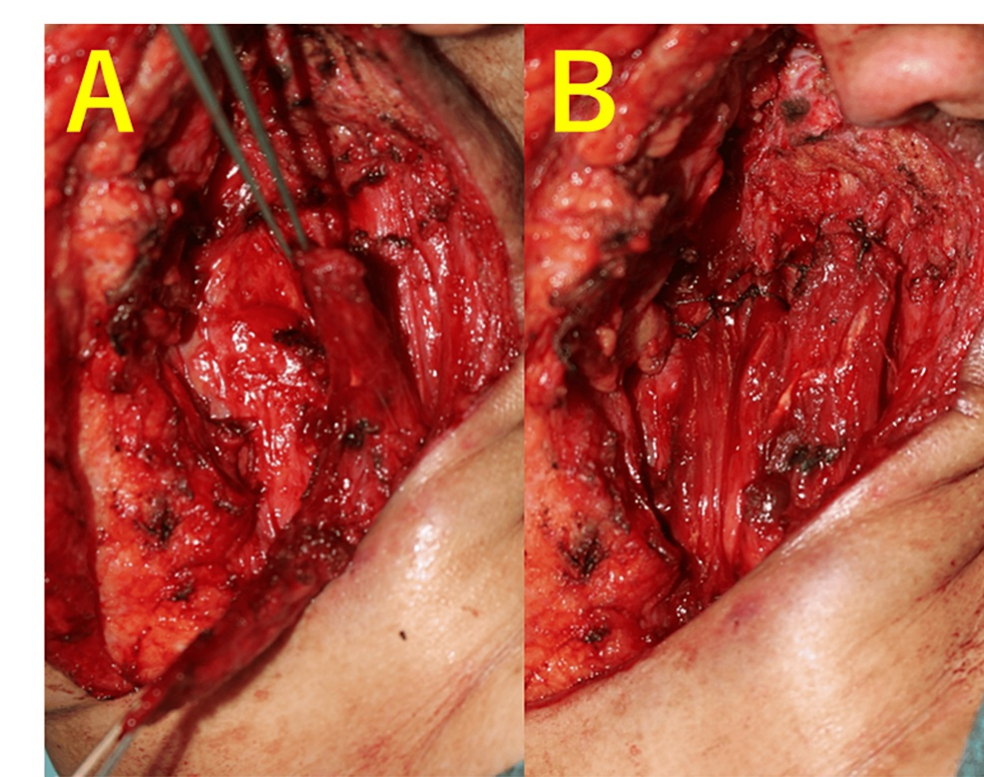
Intraoperative images (A) The SCMM flap is elevated with the middle pedicle. (B) The parotid defect is filled with an SCMM flap. SCMM, sternocleidomastoid muscle

The proximal and distal aspects of the muscle flap were sutured to the tissue surrounding the parotid defect, and the defect was filled. The wound was closed, and the surgery was completed (Figure 3). The final pathological diagnosis was a benign epithelial cyst, and no additional treatment was performed.

Postoperatively, the patient received standard wound care, infection prophylaxis, and pain management. Early mobilization was encouraged with gentle range of motion exercises for the neck starting on the first postoperative day under the guidance of a physical therapist. The patient was instructed to maintain a neutral head position and avoid excessive rotation or tilting of the head during the initial healing phase.

The patient was discharged on the second postoperative day with instructions to continue wound care and physical therapy at home. At follow-up appointments scheduled at regular intervals (one week, two weeks, one month, and three months), we monitored wound healing, assessed the improvement in torticollis, and addressed any concerns or complications.

**Figure 4:**
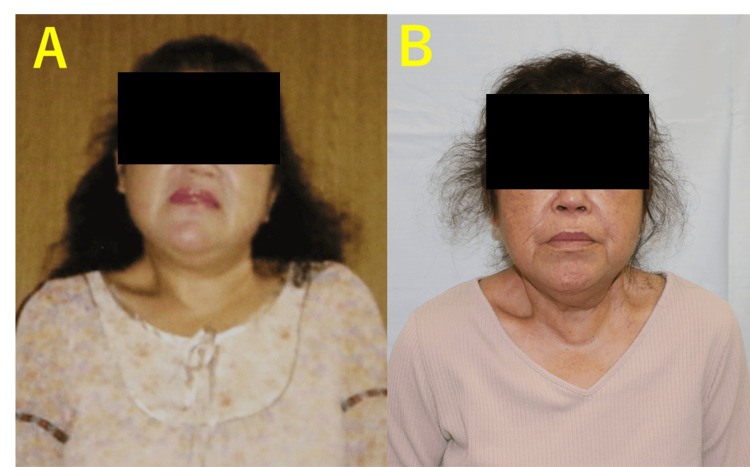
Photographs of the 64-year-old woman (A) Taken 30 years prior to surgery, showing the neck is tilted due to torticollis. (B) Taken three months after surgery, showing improvement in torticollis. The depression on the left cheek is not noticeable.

During follow-up appointments, the patient’s progress in physical therapy was evaluated, and the intensity and range of motion of neck exercises were gradually increased as tolerated. By the three-month follow-up, significant improvement in neck posture and range of motion was achieved, with no signs of recurrence or complications such as Frey’s syndrome (Figure 4).

## Discussion

We report two medically novel and important findings in this case. First, the implicated SCMM in CMT can be elevated as a muscle flap to concurrently reconstruct the defect following tumor resection and address torticollis. While there have been several reports of successful surgical treatment of neglected CMT or cases nonresponsive to physical therapy, even in adult patients [11,12], there have been no reports of using the muscle-causing CMT as a muscle flap. In the present study, the muscle tissue was checked intraoperatively, and it was determined that the amount of muscle required to be used for the flap could be elevated. The middle pedicle, which flows to the middle part of the SCMM, was identified, and ICG fluorography showed that the muscle was stained from the middle pedicle. It was reported that the middle pedicle supplies blood to the entire SCMM in cadavers [13], and we also reported an island SCMM flap technique with only the middle pedicle [14]. The muscle body was dissected in a bipolar manner. Both unipolar and bipolar dissections of the SCMM have been reported; however, no significant differences have been demonstrated [15,16]. While the intention was to fill the muscle flap, bipolar dissection permitted either end of the muscle to be maneuvered into the defect for filling. Despite improvement in the depression, the muscle mass was not sufficient compared to that of the normal muscle flap, resulting in a residual depression. We believe that this method is useful for preventing Frey’s syndrome, is effective in protecting against facial palsy, and acts as a nerve bed [10].

Secondly, the surgical treatment of neglected CMT in a 64-year-old patient was effective. Although there have been several reports of successful surgical treatment of CMT in adults [10,11], there have been no reports of CMT in individuals over 60 years of age. Previous reports have indicated that a corset is effective as a postoperative therapy following surgical treatment [17-19]. Although we could not perform corset fixation or postoperative therapy due to the patient’s refusal, improvement in torticollis was observed three months after the surgery.

## Conclusions

We made two new medical discoveries in this case: SCMM, which is involved in CMT, can be elevated as a muscle flap, and surgical treatment of CMT in older patients over the age of 60 was successful. Filling the SCMM flap into the defect after resection of the parotid tumor was considered a useful treatment that led directly to the resolution of CMT. Additionally, it resulted in the filling of the defect left by the parotid gland, which directly contributed to the treatment of both conditions. Parotid tumors in patients with CMT, as in the present case, are rare. Additionally, if they are malignant, it may be difficult to use the muscle as a muscle flap due to possible complications and the need for resection. We believe that we have demonstrated the effectiveness of the treatment for previously ignored CMT in older adults. Additionally, we believe that the number of patients with neglected and undiagnosed CMT is not small. With the availability of less invasive techniques, we may recommend the treatment of dystocia in older patients with an understanding of postoperative therapy.
